# Non-depleting energy in the museum

**DOI:** 10.3389/fpsyg.2023.1286669

**Published:** 2023-11-06

**Authors:** Sırma Seda Bapoğlu Dümenci, Neriman Aral, Figen Gürsoy, Emin Demir, Gül Kadan, Selim Tosun, Nur Sena Öz, Gökçe Hafızoğlu, Cansel Tosun, Şule Çelik, Mehmet Geçen, Özge Yelek, Seda Hepgül, Eda Özge Yazgan, Yasemin Çekiç

**Affiliations:** ^1^Faculty of Health Sciences, Tarsus University, Mersin, Türkiye; ^2^Faculty of Health Sciences, Ankara University, Ankara, Türkiye; ^3^Faculty of Health Sciences, Çankırı Karatekin University, Çankırı, Türkiye; ^4^Turkish Red Crescent, Konya, Türkiye; ^5^Graduate School of Educational Sciences, Gazi University, Ankara, Türkiye; ^6^Mineral Research and Exploration General Directorate, Ankara, Türkiye; ^7^Nursing Department, Faculty of Health Sciences, Malatya Turgut Özal University, Malatya, Türkiye

**Keywords:** early childhood, renewable energy sources, museum education, environment, family

## Abstract

The present study aimed to uncover whether the renewable energy education carried out in a museum has an impact on awareness of renewable energy and the environment among children and their parents. The study was carried out with two groups of 65 children aged 6 years and their parents (*n* = 47). The findings revealed significant differences between the pretest and posttest in favor of the pretest and between pretest and follow-up test in favor of follow-up test, but there was no significant difference between posttest and follow-up test. We determined It was observed that the children had a considerable willingness to participate in the sessions and used the names of renewable energy sources in their follow-up drawings or their remarks on the drawings. Moreover, given the parents’ statements, we discovered that the children acquired considerable awareness of the environment and efficient energy consumption and became acting more consciously toward renewable energy sources.

## Introduction

Energy may be a noteworthy starting point for achieving the objectives of environmental protection, one of the three fundamental components of sustainable development (Öymen, 2020). Due to being both complex and broad, sustainability has several goals to achieve. Increasing the demand for renewable energy, reducing carbon intensity, and ensuring green growth may be the most striking among these goals (Lu et al., 2023). The aim of promoting educational principles and priorities behind sustainable development and a better and multifaceted understanding of the problems in our increasingly global world needs to be embedded in education systems (Ocetkiewicz et al., 2017). In this respect, despite being a necessary means for the functioning of technological tools and one’s survival, wasting energy and insufficient environmental awareness have adversely affected habitats and sustainability in nature (Çukurçayır and Sağır, 2008; Korkmaz and Develi, 2012; Çetin et al., 2016; Esen and Esen, 2018). Therefore, regular and conscious use of renewable energy sources is considered essential, particularly for humans, to alleviate such adverse impacts (Karabağ et al., 2021).

In general, environmentally friendly energy sources come to mind regarding renewable energy. In general, renewable energy is associated with environmentally friendly energy sources. These sources already exist in nature; in other words, no harm is brought to nature when used. The energy generated from such sources interestingly meets contemporary needs to a great extent and can be recycled again (Koç and Kaya, 2015), which emphasizes the importance of consciously benefitting renewable energy sources (e.g., solar, wind, hydropower, geothermal, and biomass) (Karagöl and Kavaz, 2017). These sources are more or less available in every geographical region and seem totally nature friendly. Although Türkiye is geographically rich in renewable energy sources (Yılmaz, 2012), it is prudent to claim that social awareness of these sources has not yet been established. Similarly, the previous research highlights limited national and global awareness of the importance of renewable energy sources and how they are found and utilized (Tortop, 2012; Bilen et al., 2013; Saraç and Bedir, 2014; Çolak et al., 2015; Keramitsoglou et al., 2016; Cebesoy and Karışan, 2017; Çelikler et al., 2017; Cirit, 2017; Eren et al., 2017; Ali et al., 2019; Karaeva et al., 2019; Wojuola and Alant, 2019; Yıldırım et al., 2019; Ilias et al., 2020; Özyurt and Yalman, 2020; Szakaly et al., 2021). Hence, it may be claimed that awareness-raising activities on renewable energy sources should be provided from early childhood to contribute to awareness of renewable energy and protect nature.

Early childhood can be conceived of as invaluable years of human life. As a matter of fact, all kinds of habits to be taught to children in this period would also contribute to their personality traits and maintaining what they have acquired as a lifelong habit (Aral et al., 2002; Karaca et al., 2011; Ihmediah and Oliemat, 2015). Yet, despite being keen on learning, the children in this period may also need to have concrete experiences and “learning by doing” to successfully interpret and rely on their learning (Andiema, 2016; Ülker Erdem et al., 2017; Yıldırım and Özyılmaz Akmaca, 2017). In this sense, museums have recently attracted attention as educational environments where children can learn by doing. In museums, children not only have the opportunity to learn through their own experiences but also internalize what they have learned and turn it into behaviors and attitudes (Lopez Alcarria et al., 2014; Akman et al., 2015; Akgün et al., 2017; Guardino et al., 2019). The previous research also confirmed this idea and suggested that children would make the knowledge acquired through museum visits permanent (Dağal and Bayındır, 2016; Rönkkö et al., 2016; Aktın, 2017; Karakaş and Eğitmen, 2017; Dilli et al., 2018; Gong et al., 2020). In this context, it may be more convenient to introduce the abstract concept of renewable energy to children in early childhood in a practical and joyful way in a museum setting. Yet, the literature hosts a limited number of studies on teaching renewable energy to children in a natural environment in early childhood. Among these studies, Ardoin and Bowers (2020) carried out research to introduce renewable energy sources to children in forest schools and suggested that children in early childhood would develop an awareness of the sun, water, and renewable energy in natural environments. At the same time, they emphasized that children would develop environmental awareness thanks to such by the help of/via activities. In Türkiye, Dilli et al. (2018) carried out a study to introduce renewable energy sources to children aged 6 years in a museum setting and concluded that the participating children developed an awareness of renewable energy sources and the conscious use of the environment.

Parents are known to be an integral part of supporting positive developments in children in early childhood. The behaviors of parents, their children’s utmost role models in early childhood, occupy a substantial place in forming and developing their children’s habits and attitudes. Since children learn by observing their parents’ behaviors, offering programs about learning in early childhood to families is considered to be important (Ekinci-Vural and Kocabaş, 2016; Hayakawa et al., 2016; Kınık et al., 2016; Özel and Zelyurt, 2016; Boz et al., 2018; Marti et al., 2018; Puccioni et al., 2020). However, the relevant literature seems to have missed education/programs on renewable energy sources for parents, which, in turn, contributes to the importance of increasing social awareness of renewable energy sources. Given these considerations, the present study attempted to reveal whether the renewable energy education carried out in a museum and parent-oriented training have an impact on awareness of renewable energy and attitudes toward the environment among (six-year-old) children and their parents.

## Methods

### Research design and participants

We carried out this mixed-design research with children aged between 60 and 72 months, attending kindergartens and daycare centers affiliated with Mamak and Altindag municipalities, and their parents. We resorted to the opinions of teachers and administrators who knew the potential participants very well and selected the sample considering the criteria of students’ socioeconomic status (SES), special needs status, how long they had been enrolled in preschool education, and previous visits to the museum where the project would be implemented. Accordingly, we recruited a total of 112 participants in the study: 65 socio-culturally disadvantaged preschool children (35 girls, 30 boys) without any health problems who had been attending preschool education for at least 6 months and had not visited the museum before and 47 parents providing their informed consent to participate in the study with their children.

### Data collection tools

In the quantitative phase of the research, we utilized the “**Children’s Attitude toward the Environment Scale: Preschool Version (CATES-PV)**” to reveal the participating children’s attitudes toward the environment before and after the implementation of renewable energy-related activities. Moreover, we readministered the instrument 3 weeks after the implementation to determine any changes to their attitudes. The **CATES-PV** was developed by Musser and Diamond (1999)and adapted into Turkish by Gülay (2011). The scale consists of 15 items and is scored on a four-point Likert-type scale. One may obtain 60 as the highest score and 15 last the lowest score on the scale, and higher scores indicate a higher attitude toward the environment.

In the qualitative phase of the research, we deployed the “drawing,” “observation,” “portfolio,” “diary keeping,” and “photograph taking” techniques to uncover the children’s achievements in renewable energy. In addition, we recruited the participating parents for face-to-face interviews (a semi-structured interview form) before and after implementing the renewable energy-related activities to reveal the impacts of learning about renewable energy on their children’s lives. The form inquires the parents about environmental education, the way they use energy sources in everyday life, their children’s attitudes toward the environment, renewable energy sources, and the children’s knowledge of renewable energy sources. The form was finalized relying on the suggestion of field experts (two child development specialists, two preschool education specialists, and one measurement and evaluation specialist).

In the study, we designed renewable energy-oriented activities as educational events to be implemented for five full days (10.00–14.30). The training for parents was planned in three sessions, two face-to-face and one online. While preparing the mentioned educational activities and training, we considered the relevant literature and achievements and indicators in the Ministry of National Education (MoNE) 2013 Preschool Curriculum. The project team often rallied together online or face to face during the study and reviewed/updated the activities prepared for the parents and children when needed. Activities and parent training sessions were first submitted to expert opinion (specialists in child development, preschool education, parent education, energy, and curriculum and instruction) and then finalized in line with their views. We included games, language skills, STEM, and drama activities and experiments in the educational activities and utilized different methods and techniques to ensure the active participation of the children. Parent training also covered educational activities, lecturing, discussions, and demonstrations that allowed the parents’ active engagement.

### Procedure

The Scientific Research and Publication Ethics Committee of Tarsus University granted ethical approval to our study (2021/33 dated 07.28.2021). Next, we held online meetings with all team members to design the project logo and launch the website where project details and announcements would be posted. Next, we opened Twitter and Instagram accounts for the projects and shared them on the website. Then, we engaged in arrangements and took measures to prevent the distraction of the children in the project site - the General Directorate of Mineral Research and Exploration, Sehit Mehmet Alan Energy Museum.

The mission of the Sehit Mehmet Alan Energy Museum, affiliated with the MTA general directorate, is to contribute to the welfare of the country by producing knowledge in the field of earth sciences. In this regard, it is the first energy museum that introduces natural energy resources such as water, sun and wind, as well as mineral assets and helps understand their use in daily life. The exhibition areas in the energy museum include sections of renewable energy sources (wind, solar, geothermal and hydroelectric energy stands) as well as mining stands (boron, coal, etc.).

In line with the cooperation with Mamak and Altindag municipalities, we set a series of meetings with administrators and teachers through the municipality officials responsible for the nurseries and daycare centers affiliated with the municipalities. We informed the administrators and teachers about our research, obtained their verbal permission to reach the parents, and carried out some preliminary activities with children upon their parents’ written consent. Then, we took pretest measurements from the children with the CATES-PV on May 16–20, 2022 and conducted face-to-face interviews with the parents on the days of the first training session (Group 1 on May 23, 2022 and Group 2 on May 30, 2022).

As part of the project, we coordinated the safe transportation of the children to the project site with a series of meetings with the management of the mentioned educational institutions. Then, we performed the pre-determined educational activities on May 23–27, 2022 for the first group of children and on May 30–June 3, 2022 for the second group between 10.00–14.30 for five consecutive days. Meanwhile, the parents were recruited for face-to-face (2) and online (1) sessions in two groups (Group 1 on May 23, 25, 27, 2022 between 13.30–14.15; Group 2 on May 30–June 1, 3, 2022 between 13.30–14.15). Immediately after finalizing the activities for the children (Group 1 on May 27, 2022 between 13.30–14.30; Group 2 on June 3, 2022 between 13.30–14.30), we took the posttest measurements from the children in the project site and reinvited the parents to face-to-face interviews at the end of the third session of their training.

We considered precautions against the pandemic during the activities with children and parents, although its impacts turned out to perish. The project site was always disinfected using professional devices during lunch breaks. Moreover, all security and safety measures were taken within the project site. The project implementation was concluded with the help of security guards and guides in the museum. Then, we took follow-up measurements 3 weeks after the project (June 19–23, 2022) in the children’s classrooms to assess the permanence of knowledge they acquired during the project.

### Data analysis

**Figure 1:**
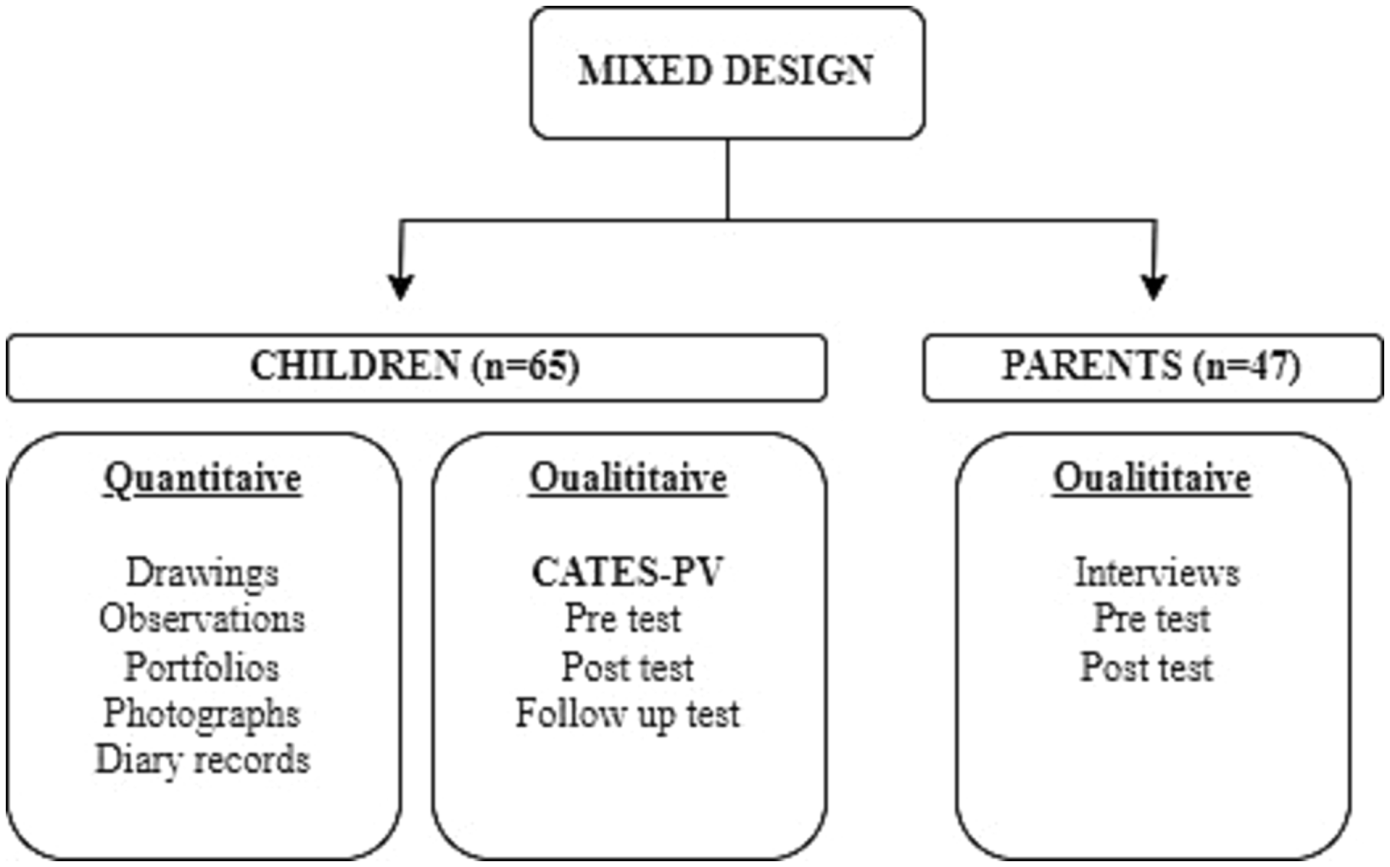
Study design.

We initially checked the normality of distribution considering skewness-kurtosis values and the results of the Kolmogorov–Smirnov test. We decided on non-parametric analyses after discovering the data to show a non-normal distribution. Accordingly, the data were subjected to the Wilcoxon signed-rank test to explore the differences between the children’s pretest, posttest, and follow-up CATES-PV scores. We performed the analysis on the SPSS program and accepted a *value of p* <0.05 as statistically significant (Figure 1).

**Figure 2:**
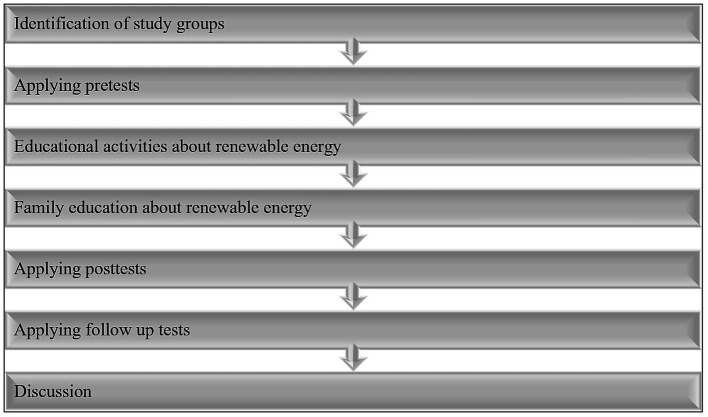
Data collection procedure.

When it comes to the qualitative data, we subjected the children’s drawings, our observations, portfolios, diaries, and photographs during the project and interviews with the parents to content analysis (Figure 2).

### Findings

**Figure 3:**
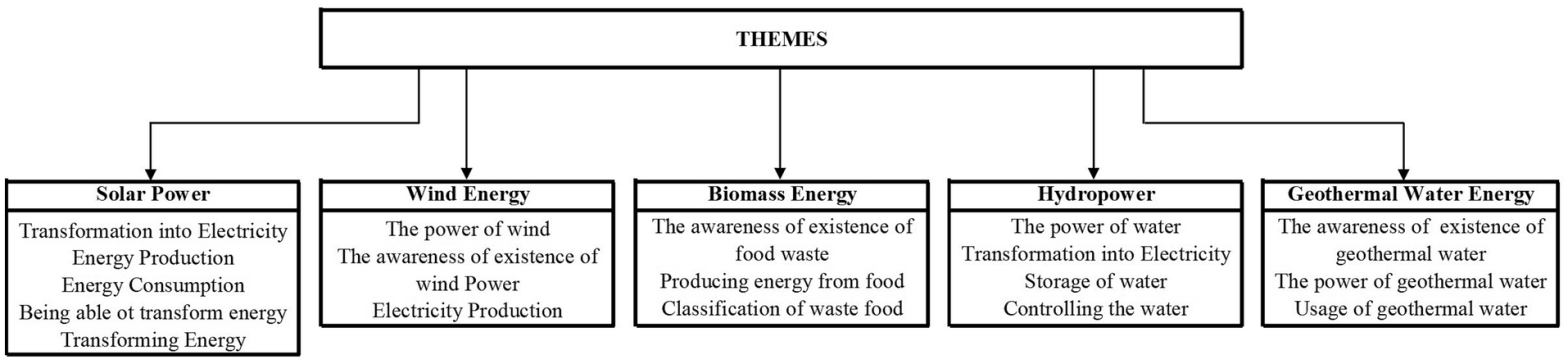
Themes of drawing analysis.

The findings of the study, performed to uncover whether renewable energy-related activities have an impact on children’s attitudes toward the environment and awareness of renewable energy, are presented below separately for the participating children and their parents (Figure 3).

#### Quantitative findings for children

The findings showed the mean pretest, posttest, and follow-up CATES-PV scores to be 49.70 ± 4.88, 51.52 ± 5, 82, and 52.71 ± 4.60, respectively. Below are the results of the Wilcoxon signed-rank test to determine the differences between the children’s CATES-PV scores (Table 1).

**Table 1:** Programme flowchart.

Day 1	Day 2	Day 3	Day 4	Day 5
Grabbing attention-A letter came from the postman	Expert opinion	Expert opinion	Expert opinion	Expert opinion
Expert opinion	The power of the wind	Playing a game “Biomass”	Playing a game “Fishing net”	Playing a game “Water transport”
The power of the sun	A trip to wind turbines	Trip to the biomass section	Building a water mill	Steam experiment
Scientific trip to solar panels	Listening to the wind	STEM organic food waste recycling machine	Trip to the hydropower section	Trip to the geothermal water section
Lunch time	Lunch Time	Lunch time	Lunch Time	Lunch time
Family education-1	STEM Boat	Learning biomass energy	Learning hydropower	Energy sources via drama
Observing the sun	Story Time Windy Day	Family education 2 online	Hydropower via drama	Family Eduation
What if the sun never set?	Kite making	Biomass collage art	Evaluation of the day	Predict, observe, explain
Evaluation of the day	Evaluation of the day	Evaluation of the day		Evaluation of the day
				Exhibition – Final Evaluation

As in Table 2, there was a significant difference between the participants’ mean pretest and posttest CATES-PV scores (*z* = −2.896, *p* < 0.05). The children’s mean CATES-PV score in the posttest (*M* = 51.52) was found to be higher than in the pretest (*M* = 49.70). To put it another way, there was a significant increase in the children’s attitudes toward the environment and awareness of renewable energy at the end of the project.

**Table 2:** Results of the Wilcoxon signed-rank test for the children’s pretest and posttest CATES-PV scores (*n* = 65).

Pretest – Posttest	*n*	Mean Rank	Sum of Ranks	*z*	*p*
Negative rank	20	27.13	542.50	−2.896	0.004
Positive rank	41	32.89	1348.50		
Ties	4				
Total	65				

We discovered a significant difference between the children’s pretest and follow-up mean CATES-PV scores (*z* = −4.226, *p* < 0.05; Table 3) and that this difference was in favor of the follow-up test. The mean CATES-PV score in the follow-up test (*M* = 52.71) was significantly higher than in the pretest (*M* = 49.70). In other words, the children maintained their awareness of the environment and renewable energy 3 weeks after the project.

**Table 3:** Results of the Wilcoxon signed-rank test for the children’s pretest and follow-up CATES-PV scores (*n* = 65).

Pretest – follow-up	*n*	Mean rank	Sum of ranks	*z*	*p*
Negative rank	19	20.58	391.00	−4.226	0,000
Positive rank	44	36.93	1625.00		
Ties	2				
Total	65				

Yet, our findings revealed no significant difference between the children’s posttest and follow-up mean CATES-PV scores (*z* = −1.799, *p* > 0.05). Accordingly, although the follow-up score (*M* = 52.71) seemed higher than the posttest score (*M* = 51.52), the difference was not significant, which implies that the project outcomes continued after the project. Therefore, we may assert that the children had similar levels of awareness of the environment and renewable energy even 3 weeks after the project (Table 4).

**Table 4:** Results of the Wilcoxon signed-rank test for the children’s posttest and follow-up CATES-PV scores (*n* = 65).

Posttest – Follow-up	*n*	Mean rank	Sum of ranks	*z*	*p*
Negative rank	22	35.07	771.50	−1.799	0.072
Positive rank	42	31.15	1308.50		
Ties	1				
Total	65				

#### Qualitative findings for children

We resorted to drawings, observations, portfolios, photographs, and diary records to present the qualitative findings for the participating children. During the implementation, the children were asked to draw pictures to describe them following the educational activities, and we noted down what the children uttered for their drawings. At the same time, we observed and kept observation notes for the children during the implementation. Along with the mentioned data, we presented findings from the portfolios, diaries, and photographs of the children below.

#### Findings of drawing analysis

In the study, we held many activities related to renewable energy sources such as solar, wind, geothermal, biomass, and hydropower. We present the children’s drawings and remarks for renewable energy sources and some examples of their drawing remarks.

##### Solar power

**Figure 4:**
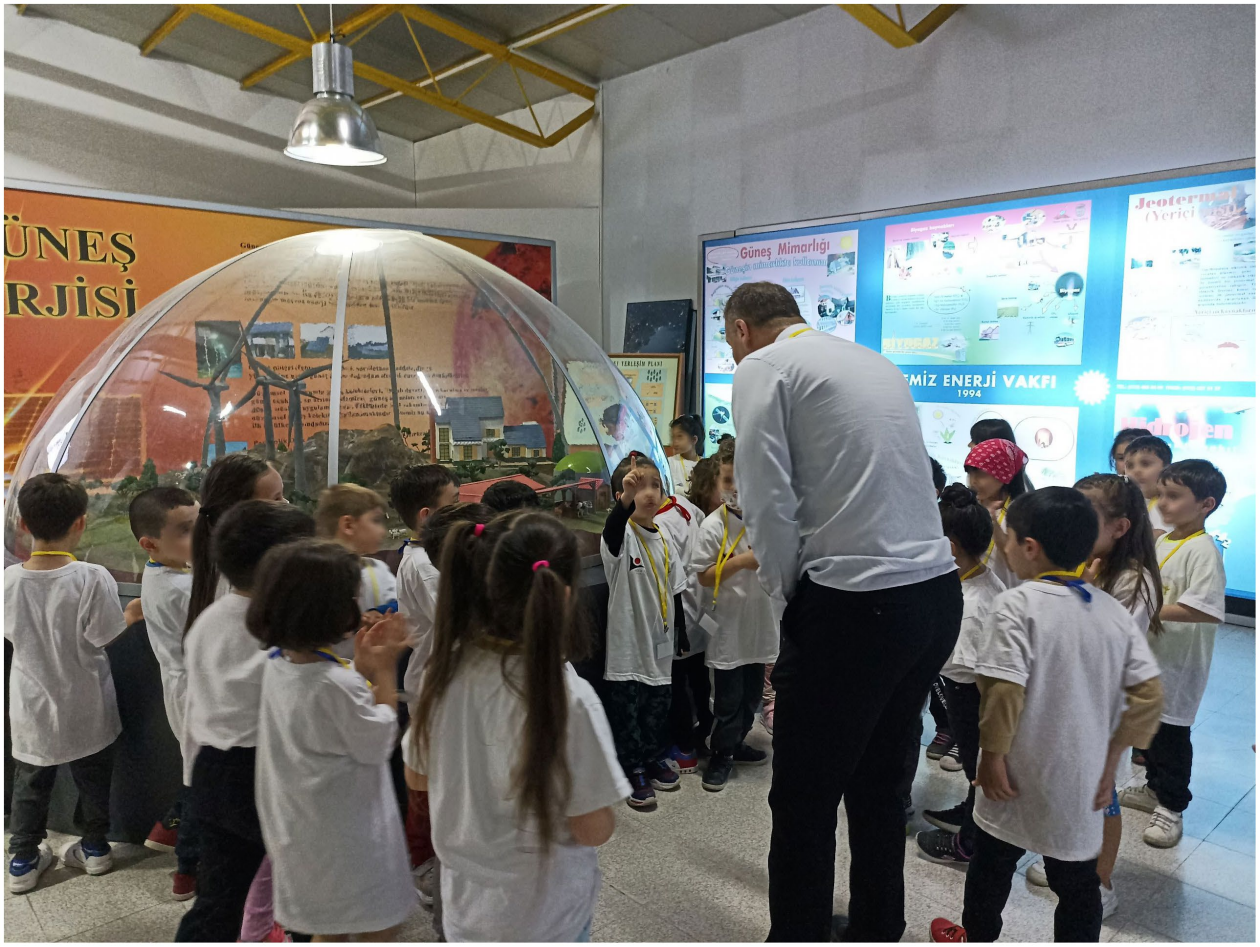
Renewable energy sources such as solar, wind, geothermal, biomass an hydropower.

**Figure 5:**
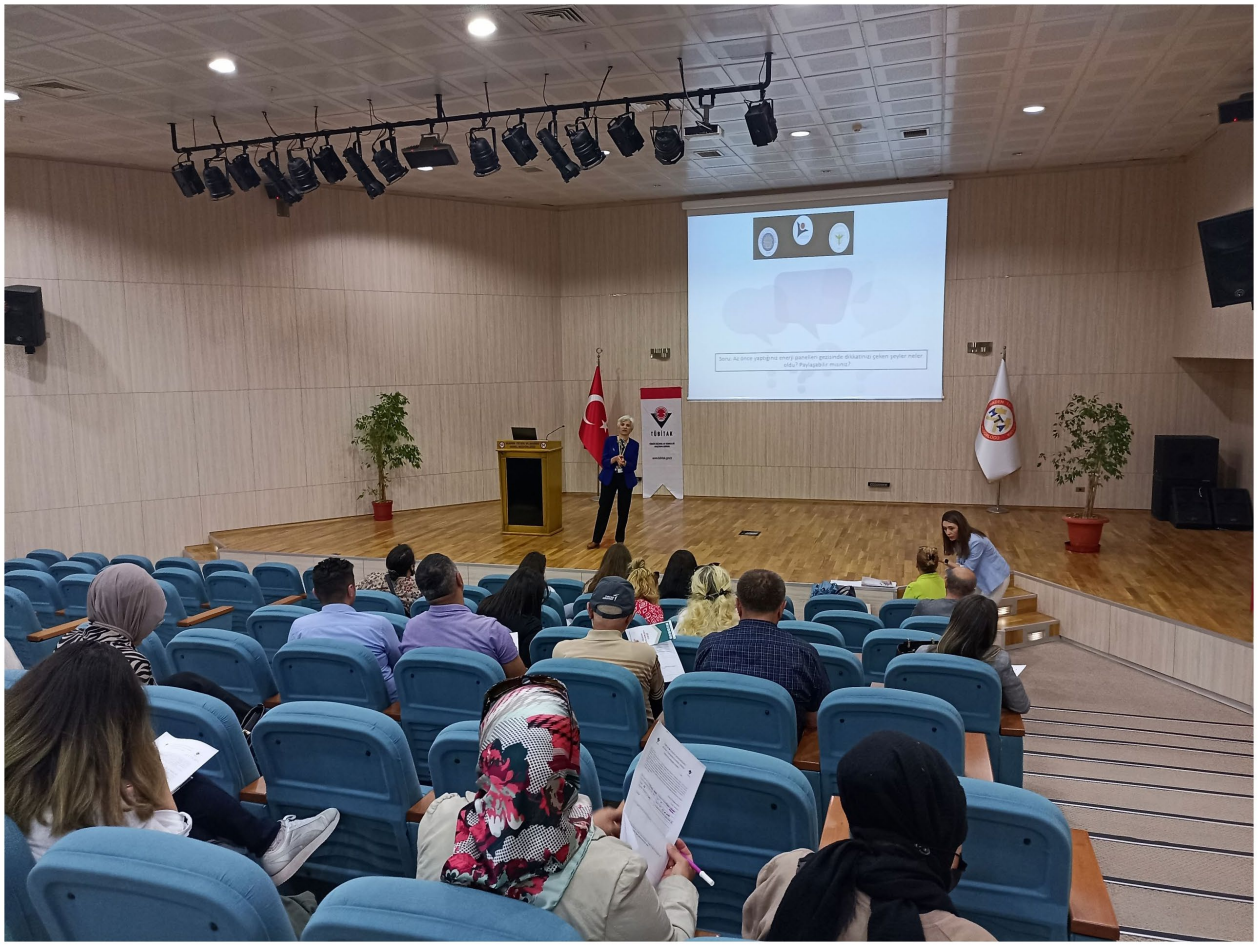
Presentation about the renewable energy sources for parents.

**Figure 6:**
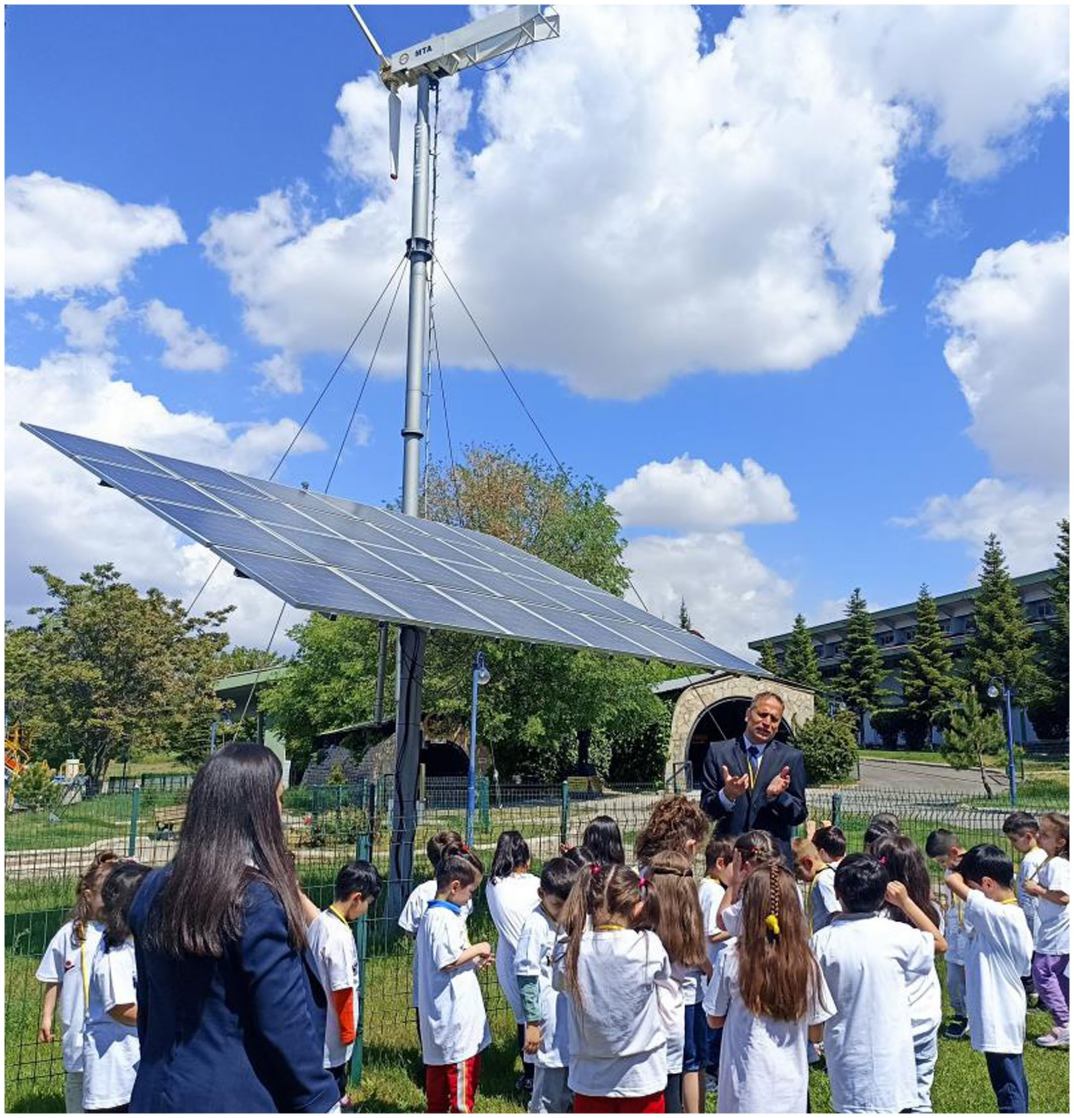
Solar power and wind energy presentation at museum.

The children’s drawings about solar energy and their remarks on their drawings may be considered important in revealing their perspectives on how solar energy is generated from renewable energy sources (Figures 4–6). Some children put the following remarks under their drawings about solar energy: “*I drew the time we stand next to a solar panel. The thing by it transfers electricity*” (C5); “*I drew solar panels and people sunbathing*” (C43), “*The purple cable is connected to the solar panel. This draws electricity and transfers it to black one. And, therefore, we can charge our phones and tablets*” (C56); “*There are trees and solar panels. There are also a TV and remote. The sun turns the TV o.*” (C31); “*One of the flowers is a rose, and the other is a daisy. Under the solar panel is a blue chair (experimental materials)*” (C9). These sample statements may imply that the children successfully reflected their awareness of solar energy in their drawings.

##### Wind energy

It seems significant that the children placed wind turbines in their drawings, and they could explain that wind turbines help generate energy with which vehicles operate and other digital transactions are carried out. The sample remarks in the children’s drawings are presented below. “*The wind makes it (wind turbine) blow. It then generates electricity. I color the sun blue because it supplies the electricity*” (C11); “*I first drew a wind turbine. For electricity…*” (C13); “*I drew a wind turbine. It spins with the wind. Phones work thanks to it*” (C57); “*The wind is coming, and a wind turbine is spinning. Warm air replaces cold air, and the wind stops*” (C41); “*When the wind comes, electricity is produced. Cars are running, the traffic lights become on*” (C38); “*Since we have learned what wind turbine is today, I drew hearts and balloons. I drew clouds and grass, too*” (C13). Their remarks and reflections imply that the children may have internalized awareness of wind energy.

##### Biomass energy

Biomass is an abstract concept among renewable energy sources that may be challenging to convey to children. However, considering their drawings and remarks, the children seemed to understand what biomass energy is. The sample remarks in their drawings are: “*If we do not throw our foods into nature and save them, we can produce electricity with these foods. No electricity means no telephone or computer. Animals may not understand how it becomes morning*” (C40); “*Dried food shells pass through a pipe and are ground*” (C12); “*I drew a television. I drew the cage in the energy room. I drew flowers and a tree, too. Also, things made of biomass…*” (C19); “*I drew daisies and sunflowers. I also drew a machine that transforms garbage*” (C3); “*Biomass is important. There are sunflowers and other materials*” (C43). These remarks indicate that the children relatively acquired and internalized the awareness of biomass energy.

##### Hydropower

We found out that the children used the concepts of rain, dam, tribune, and energy and explained what hydroelectric energy is and how it is obtained in their drawings. “*It is raining while the girl is eating a carrot. Then, electricity is generated from rain*” (C2); “*I drew a dam. Then, I drew a mill and rain. And then, I let the dam fill. When it was full, I drew a heart on the dam*” (C51); “*There are clouds. It is raining into the dam. Then, the light is on over the world*” (C19); “*I drew a dam. The water is flowing. There is something black there…tribunes*” (C37); “*I first drew rain. When hitting the ground, it becomes a pool. It then becomes a fountain and a dam later on*” (C5); “*I drew a dam. There is an anthill inside. Water comes out of the fountain to the flowers. The evaporated water is replenished with rain*” (C8).

##### Geothermal energy

The children’s drawings and their remarks on the concepts in their drawings revealed how they understood and explained geothermal energy. “*Water goes through this pipe. The sun warms it; therefore, people obtain hot water. Then, we can cook*” (C11); “*I put an egg in hot water. Then, the water becomes evaporated*” (C16); “*In my drawing, I pour hot water into the bottom of the pit to cook the egg*” (C29); “*I fill the box with hot water and cover it with a glass lid. The water has evaporated, forming drops*” (C30); “*We experimented it before. There was hot water and pots. Steam was coming out of water. Those vapors went up and became a cloud. Then, it rained*” (C44). These remarks also imply that the children relatively acquired and internalized the awareness of geothermal energy.

To sum up, we discovered that the children successfully reflected their achievements in renewable energy in their drawings, as evident in their remarks. Presenting the concept of renewable energy - an abstract topic for early childhood - with its concrete examples in the museum - an environment for children to learn by doing and experiencing - is believed to contribute to their achievements. Moreover, their changing attitudes continued even 3 weeks after the project implementation.

#### Findings of observations

In the research, we maintained observations for children throughout the activities and shared our observation notes with the project team under the leadership of the project coordinator at the end of each day. Accordingly, we deduced from the observations that educational activities significantly contributed to the participating children’s specific development areas, particularly early literacy. In the study, carrying out different activities (e.g., experiments and scientific trips) with the children allowed us to observe them in different environments. As a result of the observations, we found that the children showed a high level of participation in the activities, were not distracted during the activities, were able to solve the scientific problems raised, and were not influenced by other children visiting the museum. In addition, the children completed all activities without getting bored, and the participation rate was the highest in group work. As a result of the activities, we noticed in the children’s reflections on their drawings, their questions, and their answers to the questions raised that they got achievements in their creativity and problem-solving skills and scientific knowledge of renewable energy sources through hands-on experiences. Besides, the children’s asking what the next day’s activities would be and ability to work in harmony with their peers were among the significant outcomes of the project.

#### Findings of portfolios

As mentioned before, we resorted to different techniques to evaluate the data from the children. In this context, we also utilized portfolios generated for children. The children’s products created during the activities in the museum, including group work, were collected considering their preferences for 5 days. At the end of the fifth day, the children’s products were exhibited in a convenient venue in the museum. The children examined both their own work and the work of their friends and told their families and peers about their work, which may have contributed to their social skills. Meanwhile, by identifying the elements having attracted the children’s attention during the activities, we caught some clues about their interests and allowed them to perceive how they integrated their skills and reflected on their achievements. Besides, we discovered that one of the children created products beyond the level of development expected from their age group. Accordingly, we suggested the teachers perform detailed assessments of the child’s products.

#### Findings of diaries

**Figure 7:**
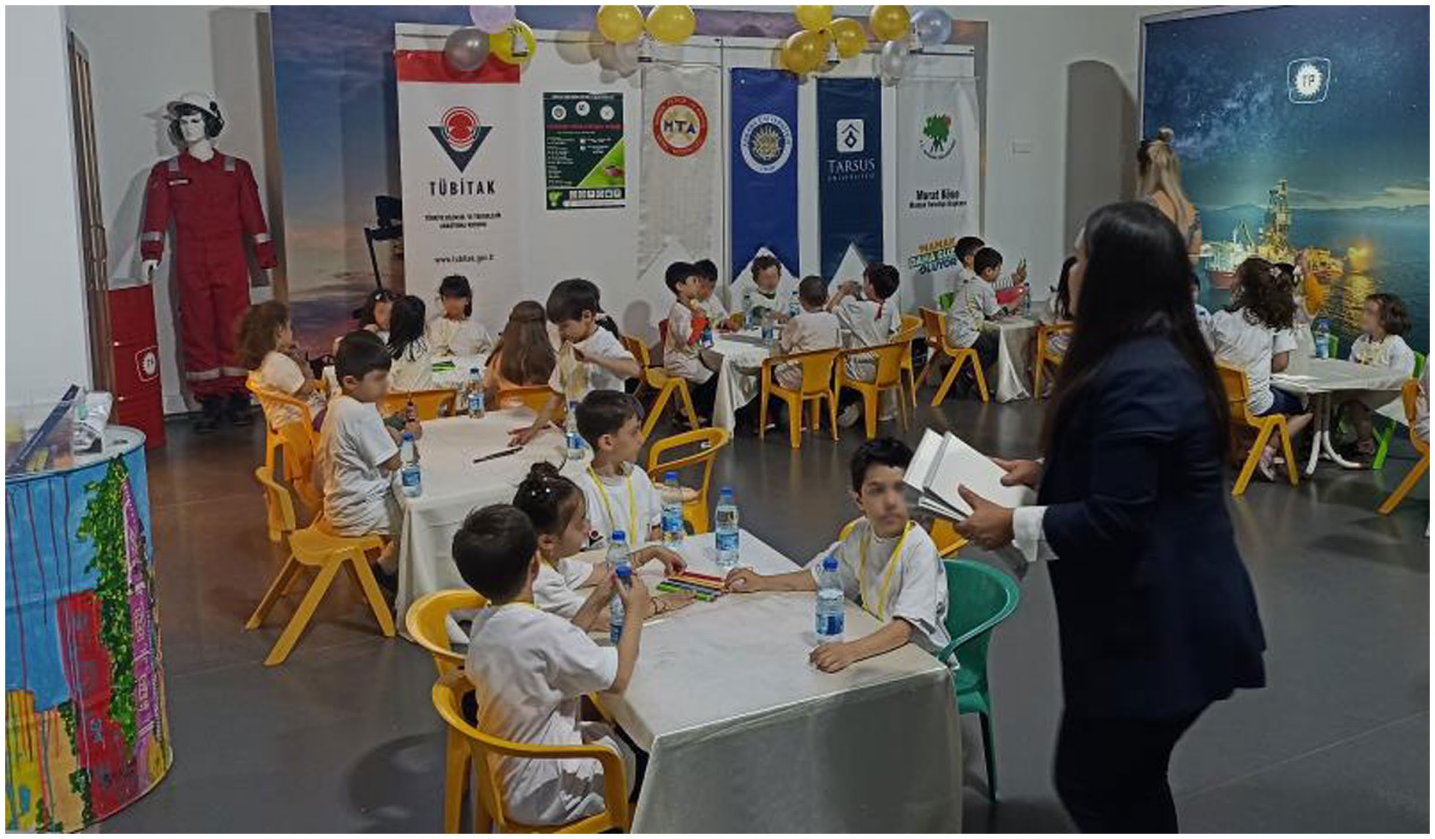
Drawing on the experiences at the museum to the diaries.

We referred to the children’s drawing skills in their diaries. In this context, we adopted the ‘draw and tell’ technique, given the children’s limited motor skills, and asked them to describe their drawings. At the end of each day, the children were given drawing notebooks and asked to draw something about the activities of the day and to describe these drawings (Figure 7). Then, the project team noted down what the children uttered about their drawings and, thus, created their diaries. Below are the sample quotes from the children’s descriptions of their drawings in their diaries: “Teacher Selim is loudly telling me about solar panels. I asked a question about two flowerpots under the sun and in the shade (experimental materials)” (C19); “When the wind comes, electricity is produced. Cars are running, the traffic lights become on” (C7); “I drew a biomass machine and its buttons. There are the heart and charge icons” (C59); “The water mill in the dam generates electricity. Water is colored blue” (C35); “They put the boiling water in the can and covered it with a plate. Then, raindrops appeared on the plate” (C27); “We learned about wind energy, hydropower, geothermal energy, solar energy, and biomass energy. I drew all the energy sources that I learned” (C53). These remarks reveal that the children were able to display the achievements they got throughout the project, which may have brought significant contributions to their areas of development.

#### Findings of photographs

Since the participating children were preschool-aged, we also utilized the photography technique among qualitative research techniques. In this regard, we analyzed the photographs of children taken during the activities throughout the project. The findings implied that the materials and scientific trip sites in the project led the children to feel surprised, happy, curious, and excited. Considering that they felt multiple emotions simultaneously, we can confidently assert the children had high learning motivation. In addition, the inclusion of game-based activities in the program entertained the children; thus, they could keep their attention on practices during the project. Implementing the program designed by field experts in a museum may suggest the effectiveness of the project on children.

#### Findings for the parents

In the semi-structured interviews held with the parents to explore their and their children’s awareness of renewable energy (Figure 5). We asked the parents before and after the project about what they understand from environmental education, how they and their children utilize energy sources in daily life, what their children do about protecting the environment, and what their children know about renewable energy (Table 5).

**Table 5:** Themes of parents interviews.

Theme	Subtheme	Some sentences
Environmental education	Awareness Protection Conscious consumption	“*Following the project, s/he has become even more conscious, and her/his awareness increased. S/he has learned how energy is generated and how wind, sun, water, and mines help generate energy*
Child behavior	Knowledge Habits Behavior	“*S/he has found the project very useful and liked it. S/he has loved the activities about watermill*”
Consumption of energy (Parent)	Habits Behavior	“*Thanks to this project, s/he has learned the types of renewable energy sources and can now distinguish them*”
Consumption of energy (Child)	Habits Behavior	“*I think the project has been effective. Children have learned about water, wind, and electrical energy. I think the examples and experiments in the project would be permanent in their minds*”
Renewable energy sources	Knowledge Awareness	**“***He sees windmills on the way back to the hometown. Now, we have told him about what they serve for. He knows it anymore”* “*S/he now knows about solar energy panels and the types of energy generated from hydropower and wind*”

We found that the parents’ responses to the first question (What do you understand from environmental education?) included common expressions, such as “**keeping the environment clean**” and “**protecting the environment**,” in the pretest and posttest. However, the posttest responses differed from the pretest responses with the expressions “**charming appearance**,” “**energy sources**,” “**the least harm to the environment**,” and “**proper use of sources**.”

The parents were also asked how their children behave toward those polluting the environment. The parents’ pretest responses to this question included the expressions “throwing the garbage away,” “getting angry,” “verbally reacting to the agent,” “taking it for recycling,” “getting upset,” and “trying not to pollute the environment.” Yet, their posttest responses did not significantly differ from their pretest responses.

The third question asked to the parents was about how they use energy sources in their daily lives. It was found out that their pretest and posttest responses shared the following expressions: “turning on the taps less,” “turning off unnecessary lights,” and “conscious consumption.” Besides, different from the pretest, their posttest responses included the expressions “placing the dishes in the dishwasher without rinsing them” and “taking care of energy-consuming needs quickly.”

Another question asked to the parents was about how their children use energy sources at home. In the pretest, parents’ responses included the expressions “turning off the taps,” “avoiding unnecessary electricity consumption,” “having sufficient awareness of consumption and acting responsibly,” “turning off the TV,” “trying not to harm the nature,” and “keeping bath time short.” In the posttest, in addition to their pretest responses, the parents mentioned that their children wanted to water the flowers with wastewater and suggested some opinions on how to manage waste.

We also asked the parents what their children knew about renewable energy sources. In the pretest, most of the parents (*n* = 20) reported that their children did not know about renewable energy sources. In the posttest, however, 19 parents suggested that their children learned and knew about renewable energy sources. Sample responses to the question are as follows: “*He sees windmills on the way back to the hometown. Now, we have told him about what they serve for. He knows it anymore* from now on (P36); “*After the project, s/he has explained solar, wind, and hydro energy at home*” (P21); “*S/he now knows about solar energy panels and the types of energy generated from hydropower and wind*” (P18); “*S/he has found the project very useful and liked it. S/he has loved the activities about watermill*” (P7); “*Thanks to this project, s/he has learned the types of renewable energy sources and can now distinguish them*” (P13); “*This project has helped my child and me. S/he can distinguish between renewable and non-renewable energy sources*” (P23); “*Actually, s/he did not know about renewable energy. S/he has learned it in this project*” (P41); “*Following the project, s/he has become even more conscious, and her/his awareness increased. S/he has learned how energy is generated and how wind, sun, water, and mines help generate energy. It has been rather useful, and I have been very satisfied with it. Thank you very much*” (P46); “*Until the last five days, s/he did not know much about renewable energy, but now s/he has learned partially about energy sources and their benefits*” (P38); “*The activities in this project have helped her/him gain knowledge about renewable energy. S/he has also kept us informed*” (P16); “*I think the project has been effective. Children have learned about water, wind, and electrical energy. I think the examples and experiments in the project would be permanent in their minds*” (P5).

We could imply from the parents’ responses that their children had an increased awareness of renewable energy, transferred the knowledge they acquired in the project to their daily lives, and shared their knowledge of renewable energy sources with others.

## Discussion

In this study, we aimed to explore whether the educational activities implemented in a museum for children and their parents have an impact on the children’s attitudes toward the environment and awareness of renewable energy. The findings revealed significant differences between the measurements taken from the children before and after the project. The significant differences in the posttest measurements were also recognized in the children’s behaviors, drawings/remarks, portfolio products, and diaries, our observations, and the interviews with their parents throughout the project. It is expected that children in early childhood may have difficulties acquiring abstract concepts. Therefore, for such children to enjoy a learning environment and internalize their learning, it may be essential to create educational settings that appeal to their multiple senses and where they are entirely active. Children assume the responsibility of learning in such environments that stimulate their inner sense of curiosity, which, in turn, contributes to their motivation (Önal and Sarıbaş, 2019). Since children are involved in educational activities in such environments where teachers act as guides, they both enjoy and internalize quite abstract concepts (Çiftçi and Uyanık, 2017). The previous research consistently highlighted the significance of using diverse methods in teaching abstract concepts to children in early childhood and reported that teachers are better to utilize experimental and drama techniques for children in this period (Gerde et al., 2013; Yalçın and Tekbıyık, 2013; İnan and İnan, 2015; Guo et al., 2016; Mavilidi et al., 2017; Tippett and Milford, 2017; Akgündüz and Akpınar, 2018). In our research, we can assert that the children’s visits to energy panels in the museum and the experiments under the guidance of educators contributed to their awareness of renewable energy sources. Their increased awareness was revealed in our observations as well as in their drawings about renewable energy sources. Based on our observations, we determined that the children actively participated in the educational activities, were excited during the experiments, and were highly motivated. In addition, the parents reported that the project was rather helpful in increasing their children’s awareness of renewable energy. Similar to our findings, Dilli et al. (2018) reported that children successfully drew renewable energy sources following energy-related relevant activities in a museum and concluded a significant difference between pretest and posttest measurements regarding the participants’ awareness of renewable energy. Besides, in the follow-up test 3 weeks after the project, we discovered that the children’s scores were still significantly higher than their pretest scores. Despite a slight decrease, there was also no significant difference between their posttest and follow-up scores. In other words, children’s attitudes toward and awareness of renewable energy continued even 3 weeks after the project, which implies that the children may have internalized what they learned in the educational activities in the museum and utilized them in their daily lives.

When learning any subject, children in early childhood may need to be able to internalize and transfer it to different domains of their lives (Breiner et al., 2012). The knowledge and skills that children would acquire in this period are likely to follow them in the long run and shape their entire behavioral repertoire (Aral et al., 2002; Hensch, 2016; Turhan and Özbay, 2016). The ability of children to internalize knowledge and behaviors without only imitating them can also be attributed to teaching methods and techniques. As a matter of fact, we can assert that the children internalized the knowledge about renewable energy during the project, where they were all active under the guidance of their teachers and were able to use it 3 weeks after the project. There are various limitations in the study. One of the limitations is that only renewable energy sources were examined in museum education. Another limitation of the study is that it was carried out only with preschool children and their parents. In addition, it is limited to the data obtained from socially disadvantaged children and families living in Ankara city centre.

## Conclusion and recommendations

Overall, our findings revealed a significant difference between the children’s pretest and posttest CATES-PV scores (in favor of the posttest) but no difference between the posttest and follow-up scores. It was also determined that the children successfully drew renewable energy sources and actively participated in the educational activities in the museum and that the parents highlighted their children’s increased awareness of renewable energy and attitudes toward the environment following the project. Based on our findings, we may recommend:

- Implementing renewable energy education to samples composed of different children in early childhood,
- Carrying out longitudinal studies to uncover the long-term impacts of the project implemented for the children, and
- Recruiting teachers for educational activities on renewable energy.

## Data availability statement

The raw data supporting the conclusions of this article will be made available by the authors, without undue reservation.

## Ethics statement

The studies involving humans were approved by the Scientific Research and Publication Ethics Committee of Tarsus University (2021/33 dated 07.28.2021). The studies were conducted in accordance with the local legislation and institutional requirements. Written informed consent for participation in this study was provided by the participants’ legal guardians/next of kin. Written informed consent was obtained from the individual(s), and minor(s)’ legal guardian/next of kin, for the publication of any potentially identifiable images or data included in this article.

## Author contributions

SB: Methodology, Resources, Supervision, Writing – original draft. NA: Funding acquisition, Investigation, Methodology, Writing – review & editing. FG: Data curation, Investigation, Project administration, Resources, Supervision, Writing – review & editing. ED: Data curation, Funding acquisition, Methodology, Supervision, Writing – review & editing. GK: Methodology, Resources, Writing – review & editing. ST: Project administration, Validation, Writing – review & editing. NÖ: Resources, Visualization, Writing – review & editing. GH: Data curation, Writing – review & editing. CT: Data curation, Investigation, Writing – review & editing. ŞÇ: Data curation, Investigation, Writing – review & editing. MG: Data curation, Investigation, Writing – review & editing. ÖY: Resources, Validation, Visualization, Writing – review & editing. SH: Resources, Validation, Visualization, Writing – review & editing. EY: Formal analysis, Investigation, Writing – review & editing. YÇ: Formal analysis, Investigation, Writing – review & editing.
